# Supplementary figures and images for: Selected by bioinformatics and molecular docking analysis, Dhea and 2–14,15-Eg are effective against cholangiocarcinoma
Source: PLoS One. 2022 Feb 3;17(2):e0260180. doi: 10.1371/journal.pone.0260180 (PMC8812988; doi:10.1371/journal.pone.0260180)

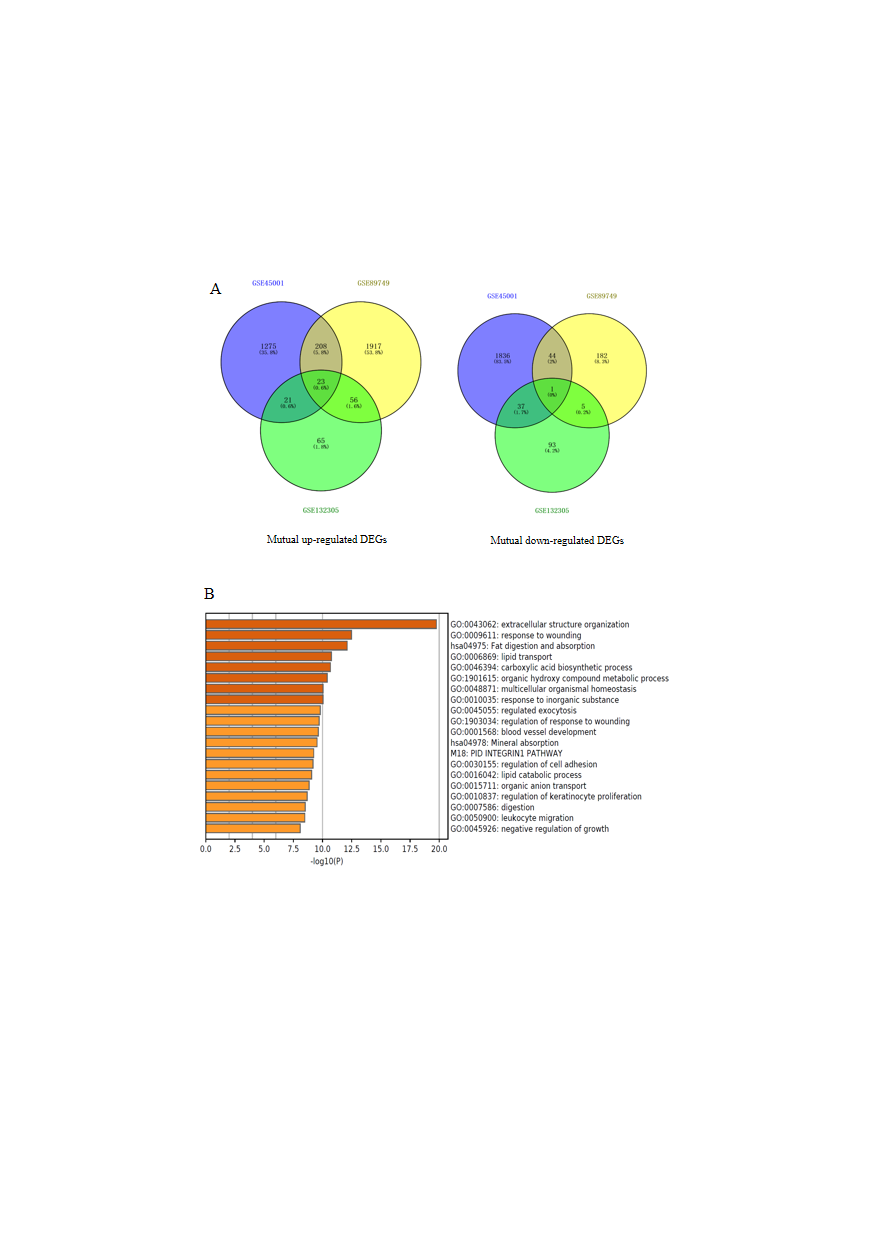

Supplement: S1 Fig — (A) Venn plot of mutual up-regulated and down-regulated DEGs among the three datasets. (B) Function and pathway enrichment of DEGs. (TIF) [file pone.0260180.s001.tif]

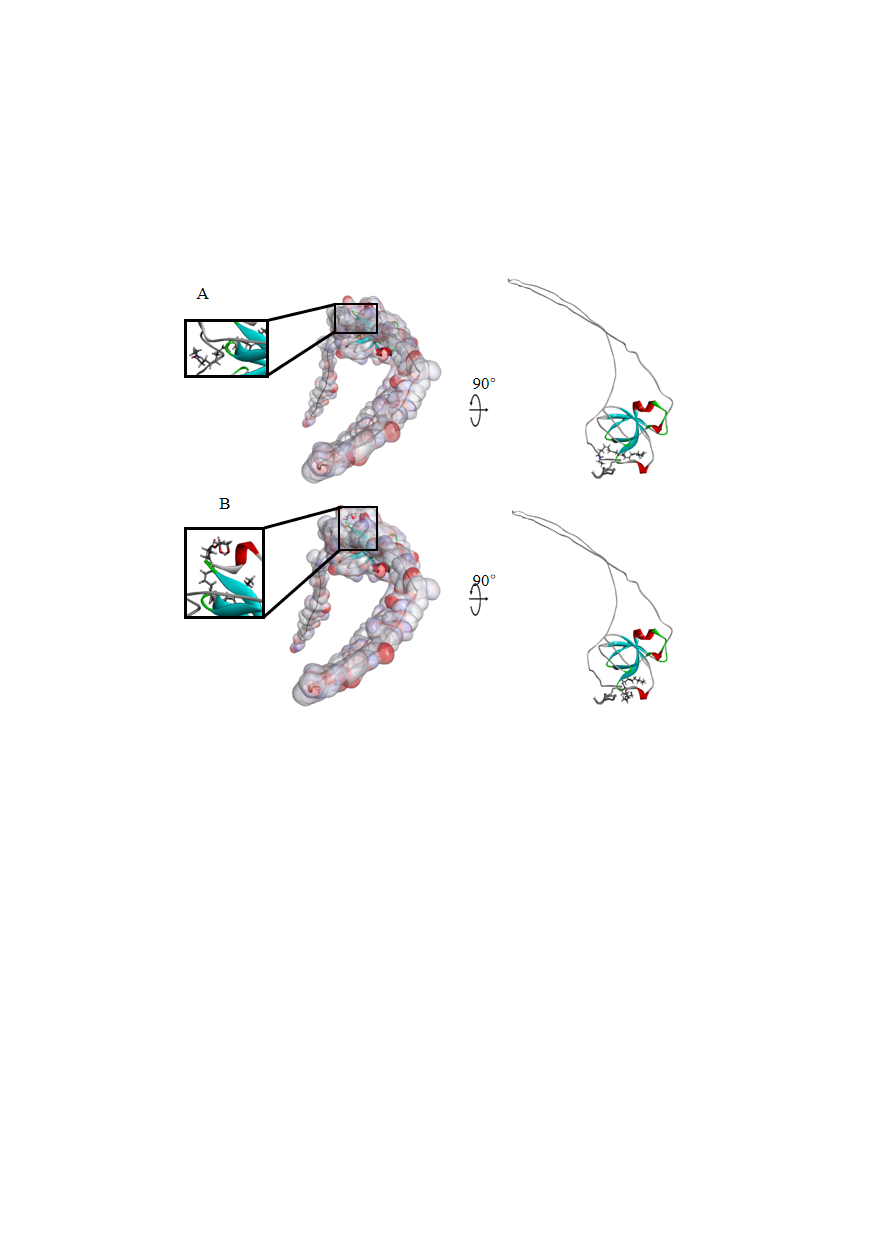

Supplement: S2 Fig — The surface of binding area was added. Blue represents positive charge; red represents negative charge; and ligands are shown in sticks, with the structure around the ligand-receptor junction shown in thinner sticks. (A) ZINC000008689961-MYC complex. (B) ZINC000027646625-MYC complex. (TIF) [file pone.0260180.s002.tif]
