# Supplementary material for: Selected by bioinformatics and molecular docking analysis, Dhea and 2–14,15-Eg are effective against cholangiocarcinoma
Source: PLoS One. 2022 Feb 3;17(2):e0260180. doi: 10.1371/journal.pone.0260180 (PMC8812988; doi:10.1371/journal.pone.0260180)
Supplement: S1 Table — (DOC) [file pone.0260180.s003.doc]

Supplementary table 1: Functional and pathway enrichment analysis of up-regulated and down-regulated genes

| Expression | Category | Term | Count | % | PValue |
| --- | --- | --- | --- | --- | --- |
| up-regulated | GOTERM_BP_DIRECT | GO:0008152~metabolic process | 14 | 4.761904762 | 4.06774E-06 |
|  | GOTERM_BP_DIRECT | GO:0006805~xenobiotic metabolic process | 9 | 3.061224490 | 4.06568E-05 |
|  | GOTERM_BP_DIRECT | GO:0048661~positive regulation of smooth muscle cell proliferation | 7 | 2.380952381 | 0.00043578 |
|  | GOTERM_BP_DIRECT | GO:0007166~cell surface receptor signaling pathway | 14 | 4.761904762 | 0.0006101 |
|  | GOTERM_BP_DIRECT | GO:0007596~blood coagulation | 11 | 3.741496599 | 0.000929258 |
|  | GOTERM_CC_DIRECT | GO:0070062~extracellular exosome | 76 | 25.85034014 | 1.11273E-06 |
|  | GOTERM_CC_DIRECT | GO:0005615~extracellular space | 43 | 14.62585034 | 1.23765E-05 |
|  | GOTERM_CC_DIRECT | GO:0005578~proteinaceous extracellular matrix | 16 | 5.442176871 | 2.1453E-05 |
|  | GOTERM_CC_DIRECT | GO:0005576~extracellular region | 42 | 14.28571429 | 0.001197897 |
|  | GOTERM_CC_DIRECT | GO:0071953~elastic fiber | 3 | 1.020408163 | 0.001422297 |
|  | GOTERM_MF_DIRECT | GO:0008201~heparin binding | 15 | 5.102040816 | 3.07492E-07 |
|  | GOTERM_MF_DIRECT | GO:0005506~iron ion binding | 10 | 3.401360544 | 0.000827323 |
|  | GOTERM_MF_DIRECT | GO:0001077~transcriptional activator activity | 12 | 4.081632653 | 0.001576362 |
|  | GOTERM_MF_DIRECT | GO:0004364~glutathione transferase activity | 5 | 1.700680272 | 0.002299304 |
|  | GOTERM_MF_DIRECT | GO:0004896~cytokine receptor activity | 5 | 1.700680272 | 0.002554495 |
|  | KEGG_PATHWAY | hsa04978:Mineral absorption | 10 | 3.401360544 | 5.61632E-07 |
|  | KEGG_PATHWAY | hsa05204:Chemical carcinogenesis | 12 | 4.081632653 | 2.00471E-06 |
|  | KEGG_PATHWAY | hsa04975:Fat digestion and absorption | 7 | 2.380952381 | 0.000249063 |
|  | KEGG_PATHWAY | hsa00982:Drug metabolism - cytochrome P450 | 8 | 2.380952380 | 0.000951463 |
|  | KEGG_PATHWAY | hsa00980:Metabolism of xenobiotics by cytochrome P450 | 8 | 2.721088435 | 0.001572278 |
| down-regulated | GOTERM_BP_DIRECT | GO:0030198~extracellular matrix organization | 10 | 12.04819277 | 2.43E-07 |
|  | GOTERM_BP_DIRECT | GO:0007155~cell adhesion | 11 | 13.25301205 | 4.01E-05 |
|  | GOTERM_BP_DIRECT | GO:0042493~response to drug | 7 | 8.433734940 | 0.002533668 |
|  | GOTERM_BP_DIRECT | GO:0030574~collagen catabolic process | 4 | 4.819277108 | 0.003044799 |
|  | GOTERM_BP_DIRECT | GO:0035987~endodermal cell differentiation | 3 | 3.614457831 | 0.006594463 |
|  | GOTERM_CC_DIRECT | GO:0005576~extracellular region | 17 | 20.48192771 | 1.72E-03 |
|  | GOTERM_CC_DIRECT | GO:0005737~cytoplasm | 37 | 44.57831325 | 1.74E-03 |
|  | GOTERM_CC_DIRECT | GO:0005654~nucleoplasm | 23 | 27.71084337 | 4.14E-03 |
|  | GOTERM_CC_DIRECT | GO:0031012~extracellular matrix | 6 | 7.228915663 | 0.010261235 |
|  | GOTERM_CC_DIRECT | GO:0000922~spindle pole | 4 | 4.819277108 | 0.012669845 |
|  | GOTERM_MF_DIRECT | GO:0005515~protein binding | 56 | 67.46987952 | 4.00E-04 |
|  | GOTERM_MF_DIRECT | GO:0005198~structural molecule activity | 6 | 7.228915663 | 0.005409325 |
|  | GOTERM_MF_DIRECT | GO:0038064~collagen receptor activity | 2 | 2.409638554 | 0.009102145 |
|  | GOTERM_MF_DIRECT | GO:0046982~protein heterodimerization activity | 7 | 8.433734940 | 0.019369645 |
|  | GOTERM_MF_DIRECT | GO:0098639~collagen binding involved in cell-matrix adhesion | 2 | 2.409638554 | 0.022602248 |
|  | KEGG_PATHWAY | hsa04512:ECM-receptor interaction | 8 | 9.638554217 | 1.06E-07 |
|  | KEGG_PATHWAY | hsa04510:Focal adhesion | 7 | 8.433734940 | 3.17E-04 |
|  | KEGG_PATHWAY | hsa04151:PI3K-Akt signaling pathway | 8 | 9.638554217 | 0.000851065 |
|  | KEGG_PATHWAY | hsa05222:Small cell lung cancer | 4 | 4.819277108 | 0.006974481 |
|  | KEGG_PATHWAY | hsa04974:Protein digestion and absorption | 3 | 3.614457831 | 0.062654101 |
|  | KEGG_PATHWAY | hsa05146:Amoebiasis | 3 | 3.614457831 | 0.086549375 |
