# Supplementary material for: Selected by bioinformatics and molecular docking analysis, Dhea and 2–14,15-Eg are effective against cholangiocarcinoma
Source: PLoS One. 2022 Feb 3;17(2):e0260180. doi: 10.1371/journal.pone.0260180 (PMC8812988; doi:10.1371/journal.pone.0260180)
Supplement: S2 Table — (DOC) [file pone.0260180.s004.doc]

Supplement table 2 Functional and pathway enrichment analysis of the modules genes

| module | Term | Count | P Value | FDR | Genes |
| --- | --- | --- | --- | --- | --- |
| module 1 | GO:0005615～extracellular space(CC) | 19 | 8.98E-17 | 1.110223 | GC, KNG1, RBP4, CRISP3, TF, OLFM4, SPARCL1, HP, TCN1, CFP, APOA4, ORM1, APOB, FGB, ALB, APOC3, LTF, GOLM1, SPP1 |
|  | GO:0070062～extracellular exosome(CC) | 22 | 1.53E-15 | 1.62E-12 | GC, KNG1, MGAT4A, RBP4, CRISP3, TF, OLFM4, SPARCL1, HP, METTL7A, CANT1, APOA4, ORM1, APOB, APOA2, FGB, ALB, APOC3, LTF, HBB, GOLM1, SPP1 |
|  | GO:0005576～extracellular region(CC) | 18 | 6.77E-14 | 7.05E-11 | GC, KNG1, RBP4, CRISP3, TF, HP, TCN1, CFP, APOA4, ORM1, APOB, APOA2, FGB, ALB, APOC3, LTF, HBB, SPP1 |
| module 2 | hsa00982:Drug metabolism - cytochrome P450(KEGG) | 8 | 2.38E-13 | 1.81E-10 | GSTM1, GSTA2, GSTM4, CYP2C19, GSTA5, ADH1B, CYP2E1, GSTM5 |
|  | hsa05204:Chemical carcinogenesis(KEGG) | 8 | 7.79E-13 | 5.93E-10 | GSTM1, GSTA2, GSTM4, CYP2C19, GSTA5, ADH1B, CYP2E1, GSTM5 |
|  | hsa00980:Metabolism of xenobiotics by cytochrome P450(KEGG) | 7 | 1.03E-10 | 7.86E-08 | GSTM1, GSTA2, GSTM4, GSTA5, ADH1B, CYP2E1, GSTM5 |
| module 3 | hsa00071:Fatty acid degradation(KEGG) | 5 | 8.25E-07 | 7.76E-04 | ACADVL, ACSL1, ACADS, HADH, ACSL5 |
|  | hsa01212:Fatty acid metabolism(KEGG) | 5 | 1.43E-06 | 0.00134 | ACADVL, ACSL1, ACADS, HADH, ACSL5 |
|  | hsa01100:Metabolic pathways(KEGG) | 10 | 6.04E-05 | 0.056796 | ACADVL, PNLIP, PLD1, ACSL1, ACADS, ALDOB, PCK2, HADH, AGPAT2, ACSL5 |
