# Supplementary material for: Selected by bioinformatics and molecular docking analysis, Dhea and 2–14,15-Eg are effective against cholangiocarcinoma
Source: PLoS One. 2022 Feb 3;17(2):e0260180. doi: 10.1371/journal.pone.0260180 (PMC8812988; doi:10.1371/journal.pone.0260180)
Supplement: S3 Table — (DOCX) [file pone.0260180.s005.docx]

Supplementary table 3 MYC targeted drugs downloaded from ZICN15 database

| Number | Compounds | Compounds name | LibDock Score |
| --- | --- | --- | --- |
| 1 | ZINC000028968107 | Cochinchinenin C | 160.151 |
| 2 | ZINC000005158610 | 2',4',5,7-Tetrahydroxy-5'-Geranylflavanone | 155.564 |
| 3 | ZINC000014946303 | Cellulose Triacetate | 152.088 |
| 4 | ZINC000013838499 | Guineensine | 152.074 |
| 5 | ZINC000040164463 | Cer | 147.314 |
| 6 | ZINC000002509755 | O-Desmethylcarvedilol | 146.569 |
| 7 | ZINC000002126785 | Umbelliprenin | 145.451 |
| 8 | ZINC000000900047 | Silica Aerogel | 144.832 |
| 9 | ZINC000008689961 | Dhea | 142.883 |
| 10 | ZINC000027646625 | 2-14,15-Eg | 141.602 |
| 11 | ZINC000004098466 | Vismione D | 141.085 |
| 12 | ZINC000002526388 | 4'-Hydroxycarvedilol | 139.53 |
| 13 | ZINC000004655034 | Zeta1-Tocopherol | 138.555 |
| 14 | ZINC000002526389 | 5'-Hydroxycarvedilol | 137.753 |
| 15 | ZINC000004654839 | Demethylphylloquinone | 137.42 |
| 16 | ZINC000001667453 | epinortrachelogenin | 136.097 |
| 17 | ZINC000002528509 | 4'-Hydroxycarvedilol | 136.09 |
| 18 | ZINC000002097863 | Nitrobenzylmercaptopurine Ribonucleoside | 136.019 |
| 19 | ZINC000015206004 | Murrayanol | 135.613 |
| 20 | ZINC000001318428 | N6-Benzyladenosine | 135.142 |
