# Supplementary material for: Selected by bioinformatics and molecular docking analysis, Dhea and 2–14,15-Eg are effective against cholangiocarcinoma
Source: PLoS One. 2022 Feb 3;17(2):e0260180. doi: 10.1371/journal.pone.0260180 (PMC8812988; doi:10.1371/journal.pone.0260180)
Supplement: S4 Table — (DOC) [file pone.0260180.s006.doc]

| Receptor | Compound | Donor Atom | Receptor Atom | Distances (Å) |
| --- | --- | --- | --- | --- |
| MYC | ZINC000008689961 | Molecule:H39 | A:ILE10:O | 1.79 |
|  |  | Molecule:H43 | A:GLN132:O | 2.34 |
|  |  | Molecule:H43 | A:GLN132:O | 2.28 |
|  |  | Molecule:H47 | A:GLN132:OE1 | 1.26 |
|  | ZINC000027646625 | Molecule:H42 | A:VAL143:O | 2.58 |
|  |  | Molecule:H37 | A:MET134:O | 1.34 |
|  |  | Molecule:H40 | A:VAL143:O | 2.68 |
|  |  | Molecule:H36 | A:LEU83:O | 1.25 |
|  |  | Molecule:H37 | A:GLU81:O | 1.97 |
|  |  | Molecule:H43 | A:TYR15:OH | 2.25 |
|  |  | Molecule:H48 | A:GLN132:O | 2.14 |

Supplementary table 4. Hydrogen Bond Interaction Parameters for Each Compound with MYC
